# Master Splinter and the challenge of personalization in education

**DOI:** 10.3389/fpsyg.2025.1549702

**Published:** 2025-04-17

**Authors:** Stefano Triberti, Raffaele Di Fuccio

**Affiliations:** ^1^Department of Psychology and Health Sciences, Pegaso University, Milan, Italy; ^2^Department of Education and Sport Sciences, Pegaso University, Rome, Italy

**Keywords:** education, personalization, comics, zone of proximal development, learning, personalized learning

Master Splinter had some of an educational challenge in front of him. After all, he was only a domestic rat who had learnt *ninjutsu* by observing his owner from a cage. When he got in contact with a mysterious substance that fell out of a truck, he found himself turned into an anthropomorphic hybrid with four youngsters to care for. Not to mention these were turtle-boys he named after Renaissance painters.

When roommates Kevin Eastman and Peter Laird started working on the idea of the *Teenage Mutant Ninja Turtles* in the early Eighties, also founding an independent comics company in their own home, they did not expect the success they got. TMNT became a favorite of millions and generated an everlasting franchise that produces comics, cartoons, toys, video games and Hollywood movies to this day.

However, if we go back to the very first issues of the comic, we may find that the rat master has something to teach us too, something deeper than martial arts.

First, the strong bond between master and students goes way beyond teaching. Already in Issue #3 published in 1985 Master Splinter is lost while escaping some killer robots, and the turtles' world falls apart. They tell their new friend April “our *father* is lost.” Here we get a showoff of each turtle's personality: Michelangelo is emotional, Donatello looks for solutions, Leonardo tries to take the lead and keep the group together, Raffaello storms out in rage (luckily everything will be ok in some issues: the turtles will recover their adoptive father when facing an alien threat that has something to do with their origins as well).

Second, as a fan-made theory that could be found in some sources online suggests, Master Splinter's educational approach is characterized by a fine-tuned personalization of the teaching materials that adapt to each student's personality… but maybe, not in the way one would expect.

Indeed, it is well known that each ninja turtle has been trained in one specific weapon. This doesn't seem very sustainable. Why did Master Splinter spend so much time training each student in a different combat expertise? Even if it is not explicit in the classic comics, we can recognize a logic in the Master's choices.

Raffaello, as the most irascible and aggressive turtle, was trained to use a defensive weapon (the *sai*); Michelangelo, disorderly and chaotic, received a sophisticated weapon that requires precision (the *nunchaku*); Donatello, intelligent and tech-savvy, was given a simple wooden staff (the *bo*); finally Leonardo, the most mature and moral and destined to become the leader, was trained in *katana*, a weapon that requires no hesitation.

Why is this kind of *personalization* so interesting? If we have a closer look, it is easy to see that the approach is exactly the opposite of simply supporting students' preferences or inclinations. Master Splinter did not give his turtles weapons that they *liked* or that matched their temperament. On the contrary, it seems that he tried to *balance* their limitations, to redirect their cognitive and emotional development by providing them with opportunities to be different.

According to some educational approaches, this may come off as authoritarian and risky: there is a general idea that students should be “welcomed as they are” and any form of education should be built on their existing predispositions.

In this path we could find the Montessori (1912) approach that focuses on a pedagogical stance based on freedom of the pupils, in the sense that children could choose activities or figure out ways to solve problems independently, albeit within a predetermined space and under the guidance of teachers (Marshall, 2017). Freedom does not mean absence of discipline, on the contrary Montessori thought that self-discipline and self-reliance could naturally emerge as a consequence of experiencing freedom within the educational processes (Lillard, 2023). In the Montessori approach teachers are the observer and they could intervene when the children ask for support. The objects or artifacts, central in this approach based on direct manipulation, are in the room at the disposal of the learner who could play, and consequently learn. This would be someway in opposition to Master Splinter, because in the Montessori approach the objects are not based on the learner. On the contrary, the learner under the supervision of the teacher is responsible for auto-selecting preferred objects and activities. Splinter operates differently: he decides how to personalize what their disciples must use, on the basis of a previous assessment of the Mutant Turtles' personality. The key lies in the challenge that stimulates learning, aligned to the problem-based learning (PBL) approach, which has extensive empirical evidence supporting its effectiveness (Demirel and Dagyar, 2016), with a particularly strong long-term impact, as shown empirically in a meta-analysis that analyzes 43 studies shows a robust positive effect from PBL on the skills of students with a ES = 0.460 (Dochy et al., 2003). Other studies demonstrate the effect on the student motivation, in line with the Splinter's approach that works on the issues rather than on the strengths, as shown in a recent meta-analysis by Wijnia et al. (2024), where is evident positive effect (ES = 0.498) on motivation. The motivation is moderated by the attitudes. Master Splinter stimulates the Ninja Turtles on the problems, knowing their attitudes and proposing the opposite to elicit a challenge.

Another example of a different conception could be found in applications of Multiple Intelligences theory (MI), originally proposed by Gardner (1983, 1993, 1999). Briefly, MI theory sustains that humans are characterized by multiple, different ways of thinking and processing information. Anyone can be naturally proficient in one or more activities that could be conceptualized as independent forms of intelligence. Over the years, Gardner and his collaborators identified a number of intelligences (logical-mathematical, logical-linguistic, spatial, bodily-kinesthetic, musical, intrapersonal, interpersonal, naturalistic, pedagogical, existential). MI theory was particularly well received in education and teaching (Nolen, 2003; Phillips, 2010). MI theory informs education in the sense that educational activities and materials should be designed to promote students' different intelligences. For example, a teacher may give students different tasks that they can perform based on individual pre-existing abilities (e.g., writing an essay vs. creating a presentation based on music or images). While MI theory applied to education and teaching has the merit of promoting personalization, diversity and inclusion, it is not exempt from critiques (Alix, 2000; Klein, 1998; White, 2005). When everyone is intelligent in their own way, it is not easy to identify deficit or plus-dotation, which is essential to recognize individual needs that should be addressed in any educational project. Secondly, it is not always clear how to take advantage of proficiency in one intelligence to improve skills in another, especially when multiple intelligences are considered autonomous and independent.

Another well-known perspective that emphasizes personalized teaching and learning comes from Carol Ann Tomlinson, who wrote *The Differentiated Classroom* (Tomlinson, 2014). The perspective outlined in the book sustains that “differentiation” is a necessary strategy to help any student to achieve meaningful learning: such differentiation should be based on a personalization model that is articulated in three dimensions, namely **contents** (teachers should adapt materials to individual students' cognitive abilities), **processes** (teaching should feature a variety of strategies and methods that could adapt to individual learning styles and rhythms), **products** (evaluation methods should be various and flexible to allow students to demonstrate what they did learn in ways that are in accordance with their predispositions).

Both these approaches are even more different from Master Splinter's approach than Montessorian views, as they rely on the belief (which can be read as more or less extreme) that education materials should be designed based on abilities students are already proficient in.

Instead, Master Splinter analyzes the specific weak points of each of his students and the areas of development that need an enhancement. This approach could be possibly better understood within the pedagogical approaches inspired by the zone of proximal development (ZPD; Newman, 1989) originally theorized by Vygotsky (1987, 1978).

Vygotsky conceptualizes the learning process as a field consisting of three zones. The innermost zone represents the learner's current competence and mastered skills, encapsulated by the statement, **“*what I am able to do alone*.”** If we move with our lens in a centrifugal motion, we encounter a zone where skills and competencies are still developing, and mastery is incomplete. This is the *zone of proximal development* (ZPD), where the learner has the potential to succeed with guidance from a mentor or through the use of specific tools, summarized with **“*what I am able to do with support*.”** Beyond this lies a zone the learner cannot yet access with their own resources, represented by the statement, **“*what I am not able to do (now)*.”** Expanding the mastery zone and strengthening competencies are necessary to shift the boundaries of the ZPD outward. In most visual representations of this model, the three zones are depicted as concentric circles or ovals, with the ZPD in the middle. This suggests that learning can progress in all directions.

**Figure 1:**
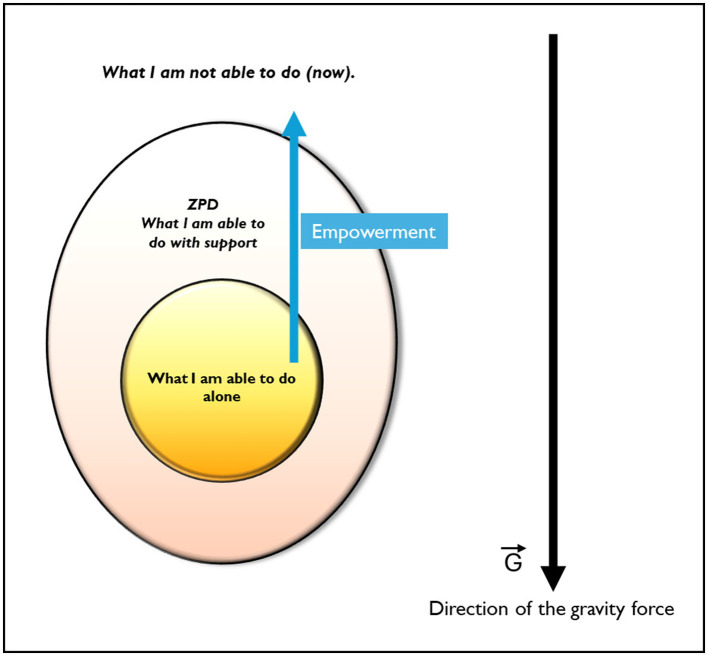
The graph depicts the three zones theorized by Vygotsky (1934, 1978) in an egg-like shape. The graph highlights the presence of the gravity force, representing that not all the directions have the same “potential”.

By extending this concept, we can imagine the model as spherical, allowing for multidirectional possibilities not limited to a flat surface (Figure 1). The concept that certain directions in ZPD models are effectively approached is similar in the representation of the “negative developmental zone,” as theorized in various studies (Poddyakov, 2006; Diaz and Hernandez, 1998). Furthermore, ZPD is strongly connected with emotions (Levykh, 2008) and its effect steer the learning. This perspective supports the ZPD as an egg-like shape, a metaphor that introduces the idea of gravity's influence on learning. Some areas of knowledge may be easier to acquire because of the learner's innate predispositions and specific orientation of their “egg” in space. In the direction of gravity, learning is naturally supported by the learner's tendencies and environment, making certain skills easier to develop. On the contrary, learning in the opposite direction of gravity requires additional effort. Aligned with this perspective, a mentor works at the edge of the ZPD, helping learners overcome their natural limitations. By providing targeted support and tools, the mentor helps the learner address areas of difficulty, counteracting the metaphorical “gravity” of their predispositions. This enables a genuine empowerment of learners, fostering their growth in areas that require deliberate effort and training. In other words, Master Splinter works on **empowerment**, focusing on the direction where the learners may face more obstacles. Apparently, this works by providing the disciples with tools that influence the direction of development as a **nudge**, in the sense that training and exercising will hopefully provide invaluable resources to discover one's own unexpected potential beyond limitations and insecurities.

This is in line with the pedagogical approach of the scaffolding, proposed by Bruner (1986) or the idea that the learning achievements could be supported by someone that assists the learner in the process. Master Splinter uses a specific scaffolding in terms of artifacts (the weapons) that are in antithesis with each personality of the turtles and, in particular, works in the opposite direction of their pre-existing attitude. The final goal is to train them in improving in an harmonious manner in all directions, both those that could be easily and naturally approached and those that are difficult to seek, taking into account the specificity of each individual and personalizing the learning. Master Splinter focuses on what learners could potentially achieve, empowering specific zones of development and trying to prevent undesirable effects and behaviors. In practical terms, Master Splinter elicits an approach that is based on facing difficulties and problem-solving based, that has a positive effect on learning performances in different contexts (Hembree, 1992; Lein et al., 2020).

Indeed, the approach sketched here is not exempt from limitations. Exactly because it calls for *personalization*, it is possible that the model should be adapted to the individual needs of learners. Personality, values, school climate for example may make some learners more or less receptive to an educational approach that could come off as relatively authoritarian (Gálvez-Nieto et al., 2022). Similarly, making learners face their own limitations may generate frustration and negative emotions (Spann et al., 2019), that deserve to be recognized and managed in order for the educational process to be a positive opportunity for growth despite obstacles.

In conclusion, the example of Master Splinter and the TMNT represents an occasion to reflect on the very meaning of **personalization of learning**, which goes beyond simply giving learners total freedom to choose how and what they would like to learn. Observing learners' personality, attitudes and predispositions is still fundamental but, at the same time, the educational path should be designed by the educator who is responsible for identifying tools and modalities that allow the learners to face their own limitations and areas of improvement.
